# Compulsory Vaccination

**Published:** 1894-06

**Authors:** B. Fincke

**Affiliations:** Brooklyn, N. Y.


### COMPULSORY VACCINATION.

B. FINCKE, M. D., BROOKLYN, N. Y.

[Read before the Homœopathic Union, April 19th, 1894.]

When Koch made his appearance on the tapis he dangerously approached Homœopathy using Isopathy for his experiments. He injected the crude poison into the system to cure it of a disease, the product of which was that poison. The German homœopaths were not slow to point this out, and forced the allopaths to withdraw their stolen thunder. They then showed by experimental cases, that, if the disease-product was potentiated, it proved not fatal as in Koch's hands but curative. Burnett followed them with his cases in the 100th potency. Now the allopaths do not claim anything for their enforced vaccination than the obscure and questionable statistics of the former and this century and the blunt assertion that vaccination protects from and modifies small-pox. But the statistics we know of and those gleaned from the cases daily occurring around us are clear and reliable and cannot be gainsaid. They say vaccination and re-vaccination has no influence upon small-pox and frequently does incalculable harm. The other great assistance to the virulators is the prejudice and long habit of the people, and its fear of a loathsome disease. The political doctor, like a bellwether, jumps over the obstacle in his way, the want of a scientific principle for his measure, and all the millions of sheep jump after him. But men should not be sheep! And doctors should not be wolves in sheep's clothing!

Now this small-pox disease which has ravaged mankind in its numerous races for thousands of years has certainly not waited for the little Commissioner of Health who with the assistance of the police without due process of law lords it over the public under the pretense of stamping it out. Not even in a small community of a house of refuge situated on an island can he stamp it out. All the inmates, many hundreds of them, have been vaccinated and re-vaccinated four or five months ago, and lo! small-pox breaks out in the midst of them, and the stamping out begins again by inoculating the nondescript animal poison into the arms and legs of the unfortunates confined to their island. "Blind unbelief is sure to err." If these men who in their arrogance and ignorance attempt so much to force their odious and despotic measures upon such a large and otherwise enlightened community as is dwelling in the free republic of the United States—if these men would not despise what the great Hahnemann had proclaimed as the immutable law of medicinal action, and would adopt his scientific as well as humane principles and rules for the prevention and cure of disease and for the conduct of a proper life, they would look down upon these present ravings in the service of ignorance and lucre with a regret as a forlorn soul upon its misdeeds at the threshold of the other world. For it is a significant fact that the old nations in the East practiced in small-pox the same doctrine which Koch revived for tuberculosis even after having gone to the same East for further information. They inoculated the lymph of the very small-pox which they wanted to prevent and stamp out. But alas! the Hahnemannian principle, which is as old as the world, was at that time as little understood as it is, with the exception of a very small minority, at the present time.

By inoculating the small-pox poison into the body of a healthy person, that person must have been much more surely affected than if it were merely exposed to sick persons at a distance or even by contact. For the life-force potent to repel the infection when operating in the latter way is not able to do so, when the crude poisonous substance is introduced into the system by a wound made for the purpose. This works the way of intoxication and produces the legitimate effects, the small-pox disease. The same is the case with Koch's prophylaxis by Tuberculin, from which many died a painful death who would have survived for more or less time.

It is very strange to find this homœopathic principle practiced so many thousand years ago with a similar persistency as the majority of the homœopathic profession considers it at the present time. So slow grind the mills of the Eternal that even after the death of Hahnemann fifty years ago, and the publication of his *Organon*, last edition, sixty years ago, his principle of Similia Similibus is not yet understood, much less accepted by our contemporaries with the exception of a very small minority. As the old Chinese and Tartars did, they acknowledge the simile, but not the minimum dose which enables the simile to be used for proving and cure. This explains why with few exceptions, perhaps, they follow the lead of that same bellwether in vaccination and otherwise, and are no whit different from the, by them, apparently much abhorred profession.

In no case it appears clearer than in this disease of small-pox, that a one-sided acceptance of this homœopathic principle is fatal to an immeasurable extent. Strange, also, it appears to be that this same principle was imported out of the far East to the far West, and there carried out with the same fanaticism and despotism as we in our times observe in the ordinances of the Boards of Health, first time in England, from which it spread over all Europe, and produced untold misery and mortality, till Jenner came, who, unconsciously it seems, acted upon the same homœopathic principle by substituting for the small-pox poison the cow-pox poison. There was indeed the same instructive action which had caused the variolar inoculation in the East, and perhaps it was nearer to Homœopathy, because the cow-pox pustule, though appearing only at the udder of the milk-cow only resembles in its formation the cow-pox pustule. But Jenner introduced his vaccination after Hahnemann discovered the homœopathic law and he had very likely no idea of the latter event when he commenced, and as far as we know he did not care for it afterward. But also the cow-pox would not do on account of the serious affections following it, and so it was abandoned for a new mode of inoculation, that of humanized cow-pox.

The pustule produced by this modified cow-pox poison was indeed similar to the small-pox pustule, and yet nobody thought of the underlying homœopathic principle. However, in the course of time, and especially through the instrumentality of the homœopathic school, the humanized cow-pox was found to transplant the germs of disease taught by Hahnemann to come from original miasms latent in the vaccinated child, and to produce all kinds of morbid affections which even proved fatal in many cases. So this mode of humanized vaccination was abandoned likewise, and led to a new mode, which can no more be called vaccination, but is simply an infection, an attempted sepsis, effected by the insertion of a virus into the wounded skin, with a view to create an eruption which is expected to protect from small-pox. This virus is manufactured in particular stables for the purpose, where young animals, such as heifers, bull-calves, nay, even steers, bulls, and milk-cows are inoculated with virus that has originated many years previously from a cow having had the cow-pox disease spontaneously, and then propagated from animal to animal for many generations. This at least is claimed by those who manufacture and sell the so-called pure animal or bovine virus. At the same time they disclaim the purity of other kinds of virus manufactured by others for various reasons. Nobody knows in this matter what virus may be used in practice, and nobody seems to care. Also small-pox virus has been inoculated into young cattle, and horse-grease and the inoculation has been repeated from one animal to another in endless succession. The inoculation of such matter of poison is the great protection claimed by the virulating authorities at the present time. If there is really any simility to the small-pox pustules in the formation of the pustules produced upon animals, it is at least not claimed by the virulators, but it certainly prevents small-pox as little as the preceding modes of inoculation, and produces all kinds of diseases, besides disposing many individuals, in times of epidemics, to be easier infected by small-pox.

Hahnemann himself seems not to have been particularly opposed to vaccination, and though he claimed it for the confirmation of his principle, he was not in love with it. For he already mentioned (*Organon*, § 46) that the cow-pox lymph "besides protective matter contained still another tinder to a general skin eruption, consisting of small dry pointed pimples on red spots, frequently mixed with red round spots accompanied with the most violent itching," which clearly points to a psoric miasm inherent in the cow. Jenner knew very well that this cow-pox lymph produced too serious effects and abandoned it for its transference to the human subject and continuation of vaccination from arm to arm.

Now what is remarkable is that the inoculation from olden times, continuing to the present, has, as said before, proceeded upon the half-understood homœopathic principle Similia Similibus, and actually amounts to the much-condemned doctrine of Isopathy. For the old Asiatics and the modern Europeans alike make themselves guilty of that pernicious doctrine, when for protection or cure of a disease they apply the same crude substance which was produced by the same disease, to the individual by inoculation. The Chinese inoculated the small-pox lymph to prevent the contagion of small-pox. The Europeans inoculated the tuberculous matter to prevent or cure tuberculosis in man and beast. This practice is nothing but pure and unmitigated Isopathy such as the humane gentleman who first spoke of Isopathy, Dr. Lux, has never claimed and practiced. He wanted the morbid products only tested and applied in highly potentiated form. He merely contributed to carry out an idea which Hering as early as 1830 (*Archiv* X, 2, p. 24) had thrown out: "Every variola, every pestilence would then in its seed produce also the preventive remedy; epidemics, hardly born could immediately be stifled, and the first patient would heal all the rest. Plague and anthrax would lose their terrors, and whatever monster of disease the East might bring forth in the future, it would carry its own remedies with it."

Hahnemann himself at the last page of the *Chronic Diseases*, has approved of the administration of nosodes in potentiated form and must shut up the objections of all those who do not want any nosode to be used, even when it has been proved *lege artis* and the symptoms obtained present as sure a guide to its application as any other remedy in the *Materia Medica Pura*. They furnish a curious instance of inconsistency when they vaccinate with a poison which has no unadulterated pedigree for protection against small-pox. What the scientific principle may be upon which they proceed is impossible to say. They must, like the allopathic school after their bragging of the science of their standpoint, fall back upon the old attitude of medicine as being not an exact science, but a mere empirical science of experience. But they must resign their place as Hahnemannian homœopathicians if thus they acknowledge the short-comings of the allopathic school. For the medicine of Hahnemann has a universal natural law for its foundation, and therefore claims the character of an exact science which can only exist upon well-known fundamental laws. If the scientists of this branch of general science are not always able to save life according to their method founded upon law and principle, it must not be forgotten that life is entertained by God Almighty alone, and can thus far only be sustained by the instrumentality of man as the powers given to him for that purpose can sustain him. But to jeopardize life, liberty, and property by a mistaken half-understood homœopathic law, a Board of Health may be empowered by a State government as we see it for the first time in this great republic, has nothing to do with the instrumentality with which God has graced man to execute the benevolent law given to the world by Hahnemann, viz., that like will be cured by like but not the same. If therefore any prevention or protection of a disease like small-pox can be had, it can only be on the same law, that the simillimum must be given in highly potentiated form, and this simillimum can be nothing else than the small-pox lymph itself in a high potency. There is no need to compel all creation to be inoculated with one poison or another. The crude notion which has taken hold of the allopathic mind from generation to generation that the medicinal agent must be injected and inoculated by breaking the skin and penetrating the fine termination of the nerves and blood-vessels and the other constituents of the cutis by force of the hypodermic syringe or lancet is a *testimonium paupertatis* for that old school in more ways than one. For they have yet to discover that there resides in every substance a medicinal force which is a force of its own relative to the living organism, and cannot be confounded with the physical and chemical forces simultaneously contained in the substance; that this medicinal force can be obtained from the crude substance of potentiation and then on application to the tongue penetrates the whole organism in the direction of its quality.

They imitate the mad dog, the snake, the Gila monster, all of which must bite in order to instill their poison into the system—that is, may pervade it in its most vital parts. Poison and cutting are the pride of the allopathic school. If they have reached an eminence in the latter branch which no century had reached as yet the contrast to their therapeutics is the more marked. Poisoning is still the rule, their dosology to give as much as will not endanger life, still too often exerts the opposite effect. The heart fails, alas! too frequently from the effects of the palliatives given to mitigate pain or induce sleep. This type of administering poisons for prevention and cure by inoculation is a humiliation to a civilized nation at the end of this century, and degrades the free people of this country to a new slavery which is as bad as any that ever was. Nobody is any more at liberty if the Board of Health, by force of an unconstitutional law, can take hold of your person to be inoculated with a poison no sane person can approve of, on penalty of forfeiture of your life, liberty, and property. You must be vaccinated in order that a person two miles off may be free from danger of contagion from small-pox. Strange that the allopathic profession always ridiculed our infinitesimals on the principle of impossibility, and now they compel everybody to submit to their authority on penalty of the law on the plea of possibility.

Is the allopathic profession going mad to claim such an enormous power over a civilized nation? Will the old adage come true, "Whom the gods would destroy they first make mad"?

If not opposed in time, compulsory vaccination will in its consequences override all the privileges and rights not only of every citizen but also those of a regularly educated and licensed physician and Homœopathy will meet its doom. Here would the often quoted sentence relating to "freedom of medical opinion and action" find its proper place for the majority of our profession to defend it against the encroachments of the allopathic profession through its political doctors.

Time is too short to point out the dangers to the people whereof its natural fear of contagion and infection is taken advantage of to abridge and destroy altogether the inalienable natural rights vouchsafed by the Declaration of Independence and the Constitution of the United States.

For the present it would suffice if means could be found to repeal the unconstitutional law of compulsory vaccination as soon as possible, and to see to it that it will not creep into the new Constitution of the State of New York at the convention soon to be held.

BROOKLYN, April 19th, 1894.

